# On the single and multiple associations of COVID-19 post-acute sequelae: 6-month prospective cohort study

**DOI:** 10.1038/s41598-022-07433-8

**Published:** 2022-03-01

**Authors:** Beatriz María Jiménez-Rodríguez, José Gutiérrez-Fernández, Eldis Maria Ramos-Urbina, Ana Dolores Romero-Ortiz, Paula Isabel García-Flores, Maria Inmaculada Santiago-Puertas, Maria José Martín-López, Genaro López-Milena, Rene Fabregas, Concepción Morales-García

**Affiliations:** 1 Department of Pneumology, University Hospital Virgen de Las Nieves, Granada, Spain; 2 Department of Microbiology, School of Medicine and PhD Program in Clinical Medicine and Public Health, University of Granada-IBS, 18010 Granada, Spain; 3 Department of Microbiology, University Hospital Virgen de Las Nieves, Granada, Spain; 4 Biosanitary Research Institute of Granada-Ibs, Granada, Spain; 5 Department of Radiodiagnosis, University Hospital Virgen de Las Nieves, Granada, Spain; 6 Department of Applied Mathematics and Research Unit “Modeling Nature” (MNat), Faculty of Sciences, University of Granada, 18071 Granada, Spain; 7 Department of Physics and Astronomy, University of Manchester, Manchester, M13 9PL UK

**Keywords:** Viral infection, Outcomes research, Scientific data, Statistics, Risk factors, Respiratory signs and symptoms

## Abstract

Medical research is progressing to clarify the full spectrum of sub-acute and long-term effects of the post-COVID-19 syndrome. However, most manuscripts published to date only analyze the effects of post-COVID-19 in patients discharged from hospital, which may induce significant bias. Here, we propose a pioneering study to analyze the single and multiple associations between post-COVID-19 characteristics with up to 6-months of follow-up in hospitalized and non-hospitalized COVID-19 patients. The cohort study was conducted from May to October 2020 at the University Hospital Virgen de la Nieves, the leading hospital assigned for patients with COVID-19 in Granada, Spain. A total of 372 and 217 patients—with 217 and 207 included in the first and second follow-up visits—were referred 2 and 6 months after diagnosing COVID-19, respectively. We find out that post-COVID-19 clinical and mental health impairment symptoms are correlated with patient gender. Logistic adjustments showed strong statistically robust single and multiple associations of demographic, clinical, mental health, X-ray, laboratory indices, and pulmonary function variables. The functional lung tests are good predictors of chest CT imaging abnormalities in elderly patients. Bilateral lung involvement, subpleural reticulum, ground-glass opacity, peripheral lung lesions, and bronchiectasis were the most common findings of the high-resolution computed tomography images. Non-hospitalized patients suffer more severe thromboembolic events and fatigue than those hospitalized.

## Introduction

Long-term effects on multiple organ systems, caused by severe acute respiratory syndrome coronavirus 2 (SARS-CoV-2)—pathogen of coronavirus disease 2019 (COVID-19)—is one of the current problems faced by patients after passing the disease^1–3^. Preliminary studies report persisting symptoms of SARS-CoV-2, such as fatigue, dyspnea, cognitive deficit, arthralgia, impaired lung functions, and abnormal chest images^4–10^. Similar persistent symptoms were reported in patients from previous coronavirus nfections—including the 2003 SARS epidemic and the 2012 Middle East Respiratory Syndrome (MERS)^11–16^—reinforcing concerns about post-COVID-19 syndrome (PCS)^17^ (see definition of PCS in materials and methods). The work of Ongsobre et al. clearly shows the persistent and prolonged effects of lung function impairment one year after acquiring severe acute respiratory syndrome (SARS1)^15^. In addition, Liu et al. point out that deterioration of pulmonary functions and quality of life may occur up to 3 years after acute infection^18^.

Due to the limited capacity of the hospitals, only a tiny fraction of people with COVID-19 are admitted to hospitals^19–22^. However, most manuscripts published—to date—only consider hospitalized patients for cohort studies, which may induce significant bias^23^. Current 12-, 6- and 3-month follow-up studies focus on persistent clinical, psychological, pulmonary function, physical problems, and chest CT imaging only for the discharged patients^8,10,24–29^. No study has yet reported the scope of post-acute sequelae of COVID-19 in a singular or multiple manners, including the non-hospitalized patients. Also, the association between pre-existing respiratory diseases and PCS is still unknown.

Our goal is to analyze the degree of single and multiple associations between clinical characteristics, demographic features, mental health, and pulmonary function test linked to PCS—of the first variant of SARS-CoV-2—in patients with/without previous respiratory diseases, hospitalized or not, and the abnormalities of chest CT images.

## Results

**Figure 1:**
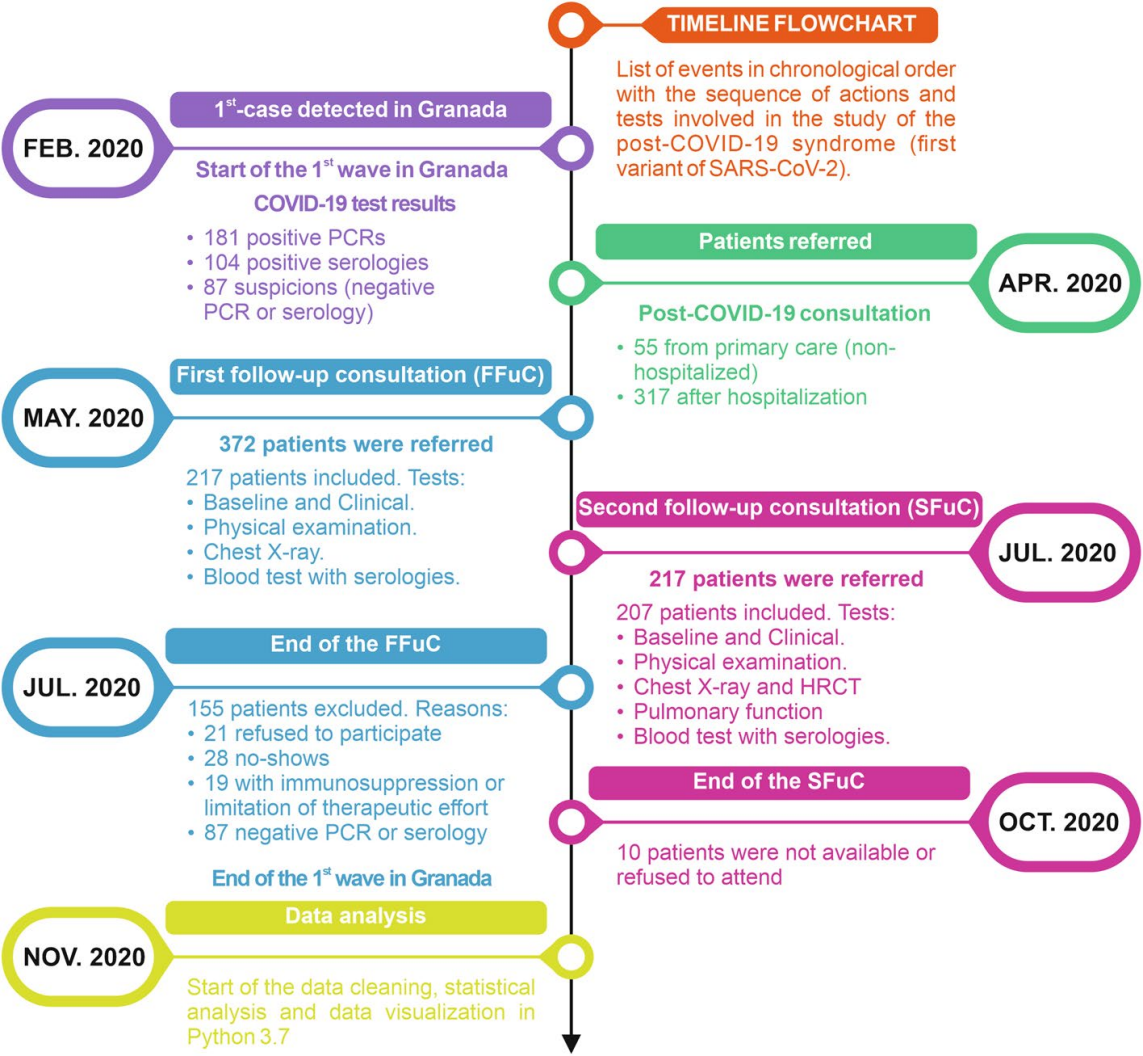
Timeline and flowchart illustrating the series of events in chronological order with the actions and tests conducted after the first case detected at the University Hospital Virgen de las Nieves. Starting with the first COVID-19 test results, the number of patients referred to post-COVID-19 consultations, the details of the first and second consultations, and data analysis.

Of the 217 patients—of which 116 (53.5%) were male—with SARS-CoV-2 was examined, including hospitalized and not hospitalized patients. These patients were monitored from May to October 2020. The follow-up study from May to October 2020 was divided into two follow-up consultations. The FFuC was carried out two months after the diagnosis of infection—from May to mid-July 2020—and the SFuC six months after the initial diagnosis—from July to October 2020. A total of 148 patients were excluded from the study for the reasons set out in Fig. 1. The median and interquartile range for age and BMI were 59 (49–68) and 28 (26–32), respectively. Active smokers or ex-smokers 89 (41%) with International Coalition Against Tobacco (ICAT) of 0[1–2]. 52.6% had been in contact with family members with suspected or confirmed COVID-19. At the FFuC, the most prevalent symptoms were dyspnea in 138 (53.6%) together with fatigue116 (53.5%), emotional affectation 117 (53.9%) and depression 124 (57.1%). In 64 patients (30.3%), the abnormal radiological findings continued. These and those with stress dyspnea were asked for chest HRCT. In the SFuC, 154 patients (73.3%) still showed symptoms or claimed to develop new symptomatology after the acute process that was not attributable to alternative diagnoses. Dyspnea 88 (42.5%), fatigue 99 (47.8%), hair loss 47 (22.7%), emotional affectation 91 (44%), and depression 45 (21.7%) were the most frequent symptoms. However, other alterations such as memory, concentration, and language deficits started to appear after the FFuC, reflecting a global cognitive deficit of up to 56 (27.1%). They expressed it as a lack of mental fluency with stuttering and "brain fog"^30^. Also, erectile dysfunction or decreased sexual appetite was present in 3 (1.4%)—not plotted. The overall results of the FFuC and SFuC are shown in Figure S1 of the supplementary material.

### Clinical outcomes and laboratory indices in female and males

**Figure 2:**
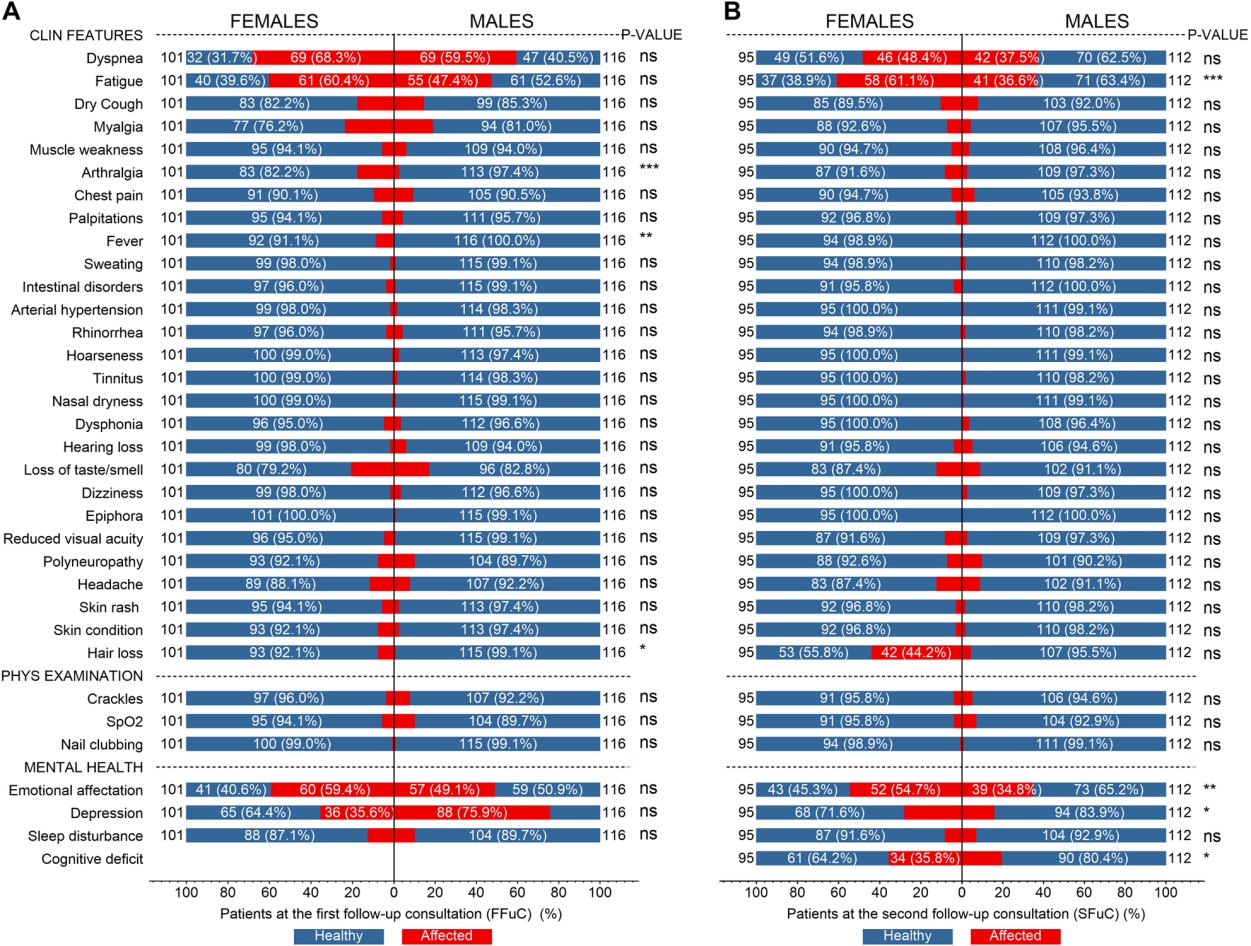
Clinical features, physical examination, and mental health of females and males with post-acute sequelae of SARS-CoV-2 for the first and second follow-up consultations—2- and 6-months post symptom onset of COVID-19. Frequencies of symptoms presented in Nº (%) of the total for each gender in the FFuC (**A**) and SFuC (**B**).

Figure 2 shows the clinical features, physical examination, and mental health of females/males with PCS—red affected and blue healthy—for first/second follow-up consultations. At the FFuC, the most frequent symptom—over 25%—in females [males] were dyspnea in 69 (68.3%) [69 (40.5%)], fatigue 61 (60.4%) [55 (47.4%)], emotional affectation 60 (59.4%) [57 (49.1%)], and depression 36 (35.6%) [88 (75.9%)], see Fig. 2A. Note here that in females, the symptoms of dyspnea, fatigue, and emotional affectation have a greater influence than in males, except for depression which affects more males than females. There was a gender difference for symptoms of arthralgia, fever, and hair loss. The rest of the less frequent symptoms—except epiphora—were still present after six months of the acute process. Figure 2B shows the results for the SFuC where the significant features for females [males] were dyspnea 46 (48.4%) [42 (37.5%)], fatigue 58 (61.1%) [41 (36.6%)], hair loss 42 (44.2%), emotional affectation 52 (54.7%) [39 (34.8%)], and depression 34 (35.8%). All these symptoms are more frequent in females than in males. From the p-values, we conclude that whether a person presents a lack of energy, emotional affectation, depression, or cognitive deficit depends on the person's gender. Also, decreased sexual appetite was observed in 1 female patient and erectile dysfunction in 2 males—not plotted.

**Figure 3:**
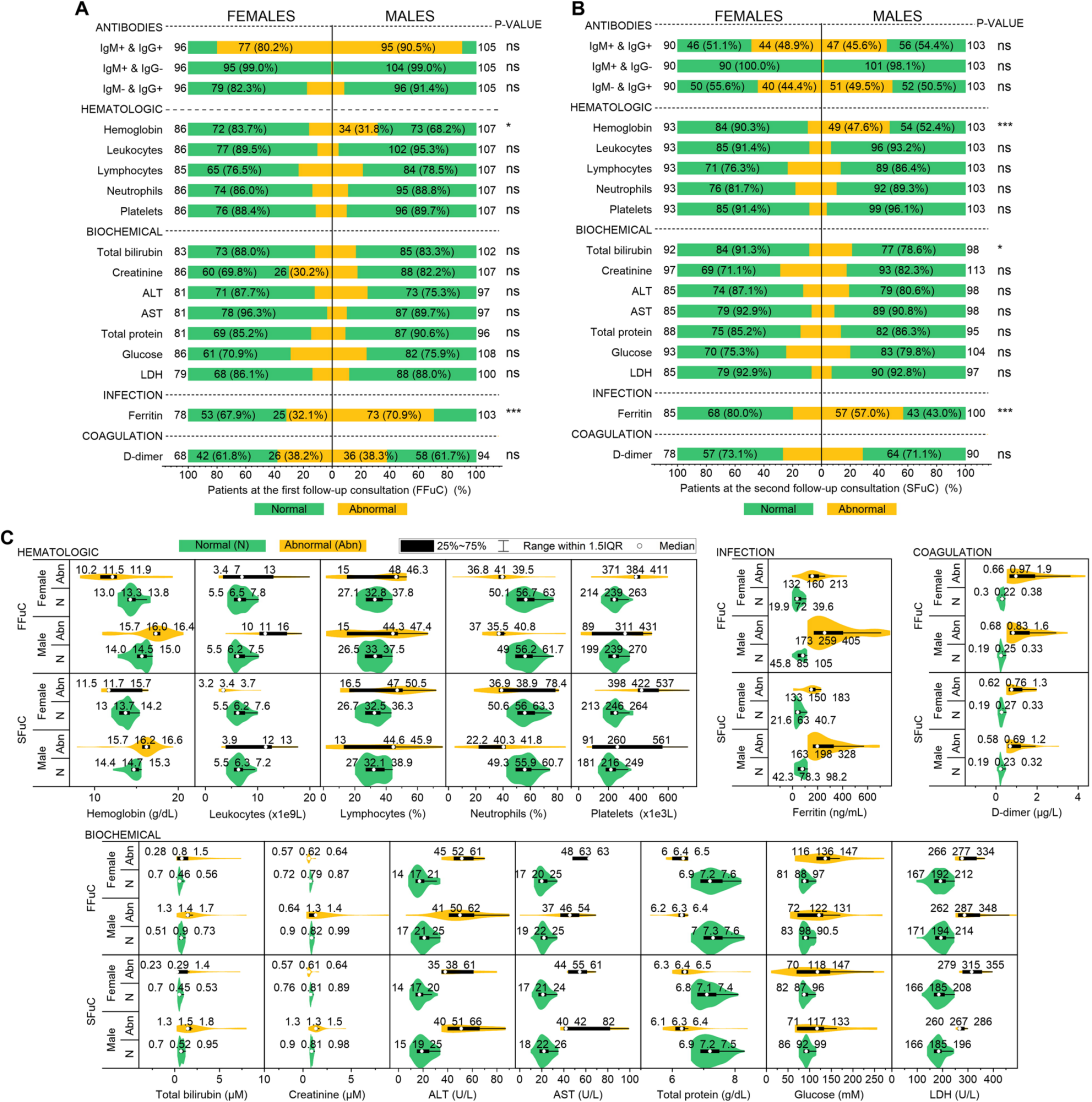
Laboratory indices of females and males with PCS for first/second follow-up consultations. Frequencies of normal (green color) and abnormal (yellow color) laboratory indices presented in Nº (%) of the total for each gender for the FFuC (**A**) and SFuC (**B**). (**C**) Violin plots show the laboratory indices' distribution, median, and quartiles for FFuC, SFuC, and gender.

The normal (N, green color) and abnormal (Abn., yellow color) laboratory indices—antibodies, hematologic, biochemical, infection and coagulation—are shown in Fig. 3 for females and males. At the FFuC—Fig. 3A, a positive IgG and IgM were presented in 77 (80.2%) [95 (90.5%)] females [males] patients. This result confirms that both women and men have passed a relatively recent infection and are developing antibodies—as expected. A negative IgG and positive IgM or a positive IgG and negative IgM were presented in less than 25% of females and males. Also, Fig. 3A,C show this cohort's most relevant abnormal indices in females [male] patients. Hemoglobin 34 (31.8%) with 16 (15.7–16.4)—men only, creatine 26 (30.2%) with a median of 0.62 (0.57–0.64), ferritin 25 (32.1%) [73 (70.9%)] with 160 (132–213) [259 (173–405)] and D-dimer 26 (38.2%) [36 (38.3%)] with 0.97 (0.66–1.9) [0.83 (0.68–1.6)]. The hemoglobin and serum ferritin indices reject the chi-square null hypothesis of independence regarding gender. The antibody test of the SFuC—Fig. 3B—shows that a positive IgG and IgM were presented in 44 (48.9%) [47 (45.6%)] females [males] patients. Also, 40 (44.4%) [51 (49.5%)] of females [males] were positive with IgG and negative IgM, e.i., the patients have been in contact with the virus and have generated antibodies after six months. Here all the frequency of abnormal laboratory indices for females is less than 25%. However, more than one-quarter of male patients had abnormal hemoglobin [49 (47.6%) with 16.2 (15.7–16.6)] and more than half abnormal ferritin [57 (57%) with 198 (163–328)]. The hemoglobin, serum ferritin indices—similar to the FFuC, and the total bilirubin reject the chi-square null hypothesis of independence regarding gender. The median and the interquartile range for all the laboratory indices for females and males are represented in Fig. 3C. Here, the abnormal values of the indices for the different genera are clearly shown.

### Association between PCS features with PRD and hospitalization

**Figure 4:**
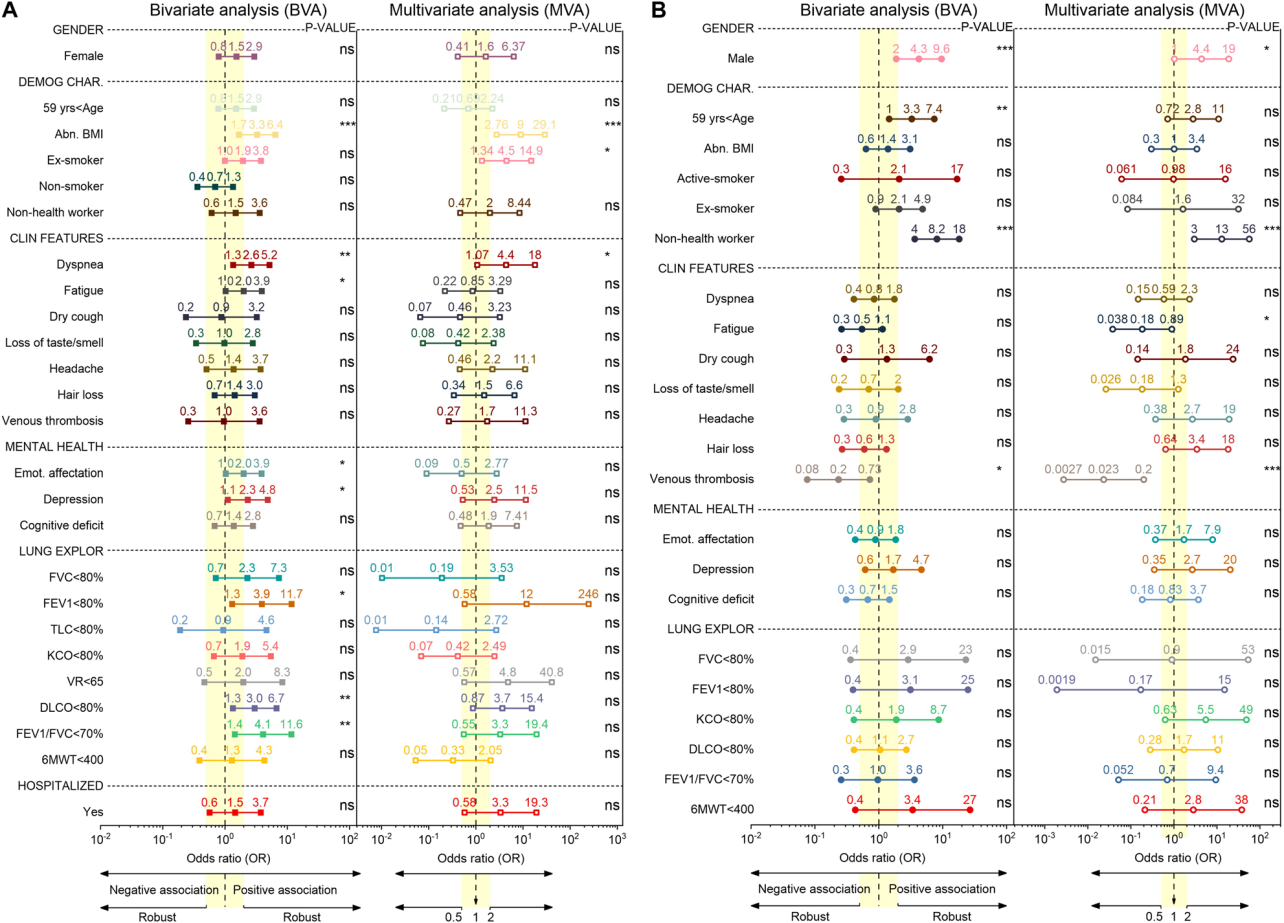
Forest plot of the odds ratio and its CIs (95%) values for the bivariate and multivariate analysis. Relationship between the PCS features with the PRD (**A**) and hospitalization (**B**). The vertical yellow band delimits the regions for a robust (OR ≤ 0.5 or 2 ≤ OR) or weak association (0.5 < OR < 2). The p values of the right column indicate the level of significance.

Figure 4 shows the OR of PCS at 6-month follow-up using BVA and MVA, given the presence of a pre-existing respiratory disease (PRD) and hospitalization. A total of 207 patients were used for the BVA of gender, demographic characteristics, clinical features, mental health diseases, and hospitalization, of which 46 had the PRD. Also, we applied a BVA for lung exploration tests in a total of 157 patients, of which 33 had a PRD. For the MVA, we used 155 patients for all predictors, of which 33 had a pre-existing respiratory disease (see Fig. 4A). In addition, two hundred seven patients were used for the BVA with the same variables—except hospitalized—used for PRD analysis, of which 173 had been hospitalized. Also, for the BVA of the lung examination tests, 130 patients—of 157—had been admitted to the hospital. The MVA included 33 patients—of 155—that were discharged from the hospital (see Fig. 4B). The descriptive analysis, medians, and IQR of all the variables are shown in Figures S2 and S3 of the Supplementary Information.

After the bivariate adjustment, the following variables are in the robust (i.e., 2 ≤ OR) range of ORs positively associated with a PRD compared to those without PRD: abnormal BMI, dyspnea, fatigue, emotional affectation, depression, impairment of FVC, FEV1, VR, DLCO, and FEV1/FVC—below their normal limits. The remaining features are in the range of less impressive OR values (i.e., 0.5 < OR < 2). Note that the highest OR corresponds to the FEV1/FVC ratio, implying that it has the highest bivalent association with the PRD compared to without PRD. Only the ORs of the abnormal BMI, dyspnea, fatigue, emotional affectation, depression, FEV < 80%, DLCO < 80%, and FEV1/FVC < 70% ratio are statistically significant (p values < 0.05). The ORs for abnormal BMI, ex-smokers, non-health workers, dyspnea, dry cough, loss of taste/smell, headache, emotional affectation, depression, all the impaired lung exploration tests, and the positive hospitalized status are in the positive or negative (i.e., 2 ≤ OR or OR ≤ 0.5) robust association range. The ORs of the remaining predictors are in a non-robust range. Statistically significant associations were found for abnormal BMI, ex-smokers, and dyspnea, as shown in Fig. 4A.

After the BVA, in the range of robust OR scores positive associated to hospitalized compared to non- hospitalized patients are the following features: male sex, age > 59 years, active-smoker, ex-smokers, non- health worker, the impairment of FVC, FEV1, and 6MWT. Venous thrombosis is the only predictor with a negatively associated with hospitalized patients. The explanation for this result is that pharmacological thromboprophylaxis was provided during hospitalization. The remaining variables after the BVA are in the less impressive range of ORs, implying a negative association with hospitalized patients compared to non-hospitalized patients. Statistically significant associations were found for gender, age > 59 years, non-health workers, and venous thrombosis (see Fig. 4B). After the MVA, the values of ORs for the male sex, non- health workers, age < 59 years, fatigue, loss of taste/smell, headache, hair loss, venous thrombosis, depression, impairment of FEV1, KCO, and 6MWT are in the range of strongly associated scores to the hospitalization status. The remaining variables are in the interval of non-robust OR values. Predictors of males, non-health workers, fatigue, and venous thrombosis contribute significantly to the MVA (see Fig. 4B).

### Association between PCS features with chest CT scan findings

**Figure 5:**
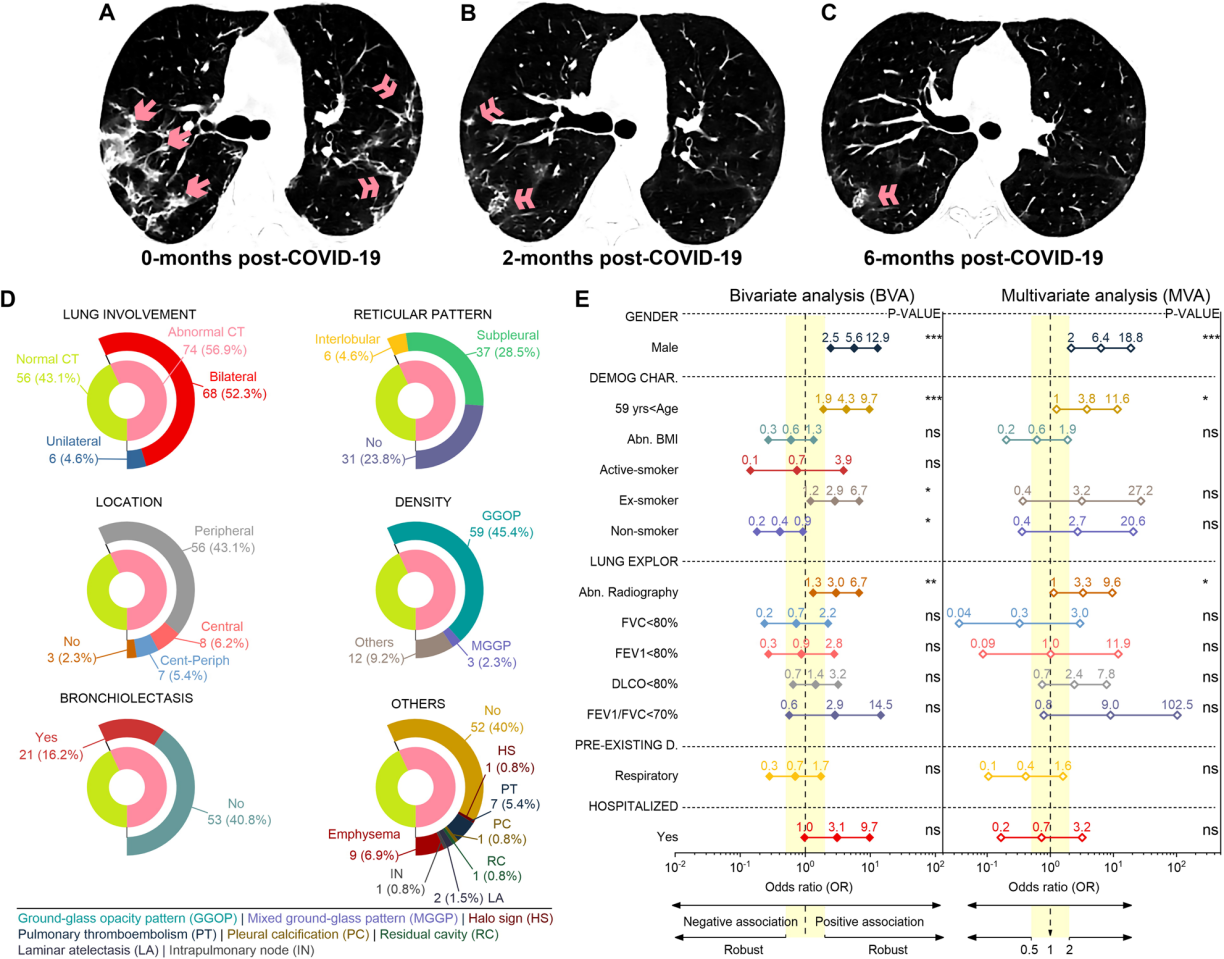
Evolution of CT scans at the same level for one patient at 0 (**A**), 2 (**B**), and 6 (**C**) months post-COVID-19. The predominant imaging pattern was the GGOs (single arrow) and reticular (double arrow). (**D**) Chest CT scan findings in all patients at the 6-month follow-up. (**E**) Forest plot of the odds ratio and its CIs (95%) values for the bivariate and multivariate analysis. Relationship between the PCS features with the normal/abnormal chest CT outcome.

The evolution of CT scans at the same level for a patient—a 65-year-old woman—at 0, 2, and 6 months after COVID-19 is depicted in Fig. 5A–C, respectively. At 0-months (Fig. 5A), the patient had patchy and bilateral ground-glass opacities (single arrows) and intralobular reticular pattern of peripheral subpleural distribution (double arrows). 2-months after COVID-19 (Fig. 5B), lesions have been significantly reduced but still have some residual reticular lesions (double arrows). Figure 5C shows the persistent subpleural banded reticular lesions (double arrow) in posterior segments of inferior lobes after 6-months of follow-up. See Figure S4A–D of the Supplementary Information for additional lung lesions from CT images of 4 patients.

Figure 5D shows the chest high-resolution CT findings of 130 patients, of which 74 (56.9%) presented chest image abnormalities due to consequences of COVID-19. The most notable—over 25%—features were: 68 (52.3%) had bilateral pulmonary involvement, 37 (28.5%) showed a subpleural reticular pattern, 56 (43.1%) had a peripheral distribution, and 59 (45.4%) exhibited a ground-glass opacity pattern. Less frequent CT findings were: unilateral lung involvement and interlobular reticular in 6 (4.6%) patients, central (8 [6.2%]) and central-peripheral (7 [5.4%]) lesion location, mixed GGO (3 [2.3%]), bronchiectasis (21 [16.2%]), emphysema (9 [6.9%]), PE (7 [5.4%]), laminar atelectasis (2 [1.5%]), and halo sign, pleural calcification, residual cavity, intrapulmonary node in 1 [0.8%] patient (see Fig. 5A).

Figure 5B shows the odds ratio after bivariate and multivariate logistic adjustments to measure the association of gender, demographic characteristics, lung examination, pre-existing diseases, and hospitalization status with the abnormal CT outcome. A total of 109 patients—of 130—were used for the BVA and MVA, of which 62 had abnormal CT results. The descriptive analysis, medians, and IQR of all the variables are shown in Figure S4 of the supplementary document. In bivariate analysis, males, age > 59 years, ex-smokers, abnormal radiography, impaired FEV1/FVC ratio, and a positive hospitalized status show a robust positive association (i.e., OR > 2). Non-smokers exhibit a robust negative OR, and the remaining variables are in the range of less impressive OR scores. Male sex, age > 59 years, non-smoker, ex-smoker, and abnormal radiography are statistically significant. Here the relationship of each characteristic is calculated separately with the CT results. After MVA, the ORs for the male sex, age > 59 years, former smoker, non-smoker, abnormal radiograph, DLCO < 80%, and FEV1/FVC < 70% in the robust interval of positively associated values. In addition, FVC < 80% and pre-existing respiratory disease are robustly adversely correlated with an abnormal CT outcome. The ORs for the remaining characteristics are in the weak correlation range. Male sex, age > 59 years, and abnormal radiography were statistically significant.

## Discussion

We have presented quantitative analyses demonstrating the presence of PCS in patients at 6-month follow-up. We started with a descriptive analysis of the clinical, mental health, physical examination, and laboratory indices findings. We have shown that the most prevalent characteristics are: dyspnea, fatigue, dry cough, loss of taste/smell, headache, hair loss, emotional affectation, depression, and cognitive deficit for clinical and mental health findings; and hemoglobin, lymphocytes, neutrophils, platelets, total bilirubin, creatine, ALT total protein, glucose, LDH, ferritin, and d-dimer for abnormal laboratory indices. This agrees with findings from previous long-term follow-up studies of SARS^11,13^ and MERS^31,32^. In addition, we have shown that fatigue, arthralgia, fever, hair loss, emotional affectation, depression, cognitive deficit, hemoglobin, total bilirubin, and ferritin associated with PCS depend on the patient's gender. In particular, the female sex is favorable for the persistence of symptomatologies—matching prior findings^33^.

At 6-months of follow-up, we found that the previous respiratory diseases and hospitalization status are strongly associated with specific demographic characteristics, clinical symptoms, mental health, and pulmonary function tests based on single and multiple PCS features. We found that PFTs were affected in patients with or without PRD—whether hospitalized or not-during the acute viral process and pulmonary involvement with restrictive and obstructive patterns and impaired diffusion capacity. The most frequent parameter to be highlighted was the diffusion capacity impairment. Its decrease may suggest an incipient DILD or the presence of pulmonary vascular abnormalities secondary to COVID-19. The bivariate analysis demonstrated the robust statistically significant association of patients with previous respiratory diseases with the following essential features: abnormal BMI, as a demographic characteristic, dyspnea and fatigue as clinical features, emotional affectation and depression as psychological complications, and impaired of FEV1, DLCO, and the FEV1/FVC ratio (i.e., positive diagnostic of obstructive and restrictive lung disease). However, after using more than one independent PCS feature, only the abnormal BMI, ex-smoker, and dyspnea had a robust statistically significant association to patients with PRD. This result implies that patients with PRD with one or more PCS features need to be monitored on a long-term follow-up basis. The following characteristics were obtained from the bivariate analysis for hospitalization status with a robust association and significant relationships: male sex, older than 59 years old, non-health worker, and venous thrombosis. Nevertheless, after the multivariate analysis, the robustness of the association for the predictors increased—except for the age > 59 years, adding fatigue as a new significant characteristic. The influential negative association of thromboembolic events—OR ≪ 1—and fatigue suggests that patients without hospitalization also need long-term follow-up.

Our findings show the positive association between abnormal BMI and hospitalization patients for COVID-19—matching previous results^34^, adding the new association with the presence of PRD. Regards to this, there is a debate on changing the relationship on BMI fluctuation of patients with overweight or obesity hospitalized for COVID-19 during their follow-up. We claim that nutritional management strategies^35,36^ during hospitalization and after discharge must be implemented to improve short- and long-term follow-up outcomes considering the comorbidities—as Di Filippo and colleagues proposed^34^.

The major strength of our study is the long-term follow-up of patients with the examination of all patients reported at 2 and 6 months, including hospitalized and non-hospitalized patients. This study is the first to present single and multiple characterizations of the long-term sequelae of COVID-19. Moreover, our study is, to date, one of the most detailed and most prolonged follow-up studies of post-COVID-19 patients. However, the greater willingness of symptomatic patients to participate in a follow-up study is a possible biasing factor—as in all observational studies. The study findings may be limited due to the single-center, nonblinded, and nonrandomized design. We understand this potential localization bias.

## Conclusions

At 6-months follow-up, PCS characteristics fatigue, arthralgia, fever,breathlessness, emotional disturbance, depression, cognitive deficit, hemoglobin, total bilirubin, and ferritin are correlated with the gender of the patient. Patients with previous respiratory diseases and abnormal body mass index, ex-smoker, and dyspnea had a robust statistically significant association. Non-hospitalized patients may suffer more severe thromboembolic events and fatigue than hospitalized patients. Functional lung tests are good predictors of chest CT imaging abnormalities in elderly patients with PCS.

The preliminary study presented here can be extended in several ways. First, the study can be prolonged to 12, 24, and 36 months of follow-up. This will enable us to study the long-term effects of PCS and define different degrees of severity. Second, adding new variables to the study will allow us to create models to predict the most frequent symptoms for medical treatments. In fact, we are working to improve our study in these directions.

## Materials and methods

### Deffiniton of post-COVID-19 syndrome (PCS)

Signs and symptoms that develop during or after an infection consistent with COVID-19, present for more than 12 weeks, and are not attributable to alternative diagnoses—following the guideline of the National Institute for Health and Care Excellence (NICE), the Scottish Intercollegiate Guidelines Network (SIGN), and the Royal College of General Practitioners (RCGP)^17^.

### Study population

The prospective cohort study was conducted at the University Hospital Virgen de la Nieves, one of the hospitals assigned for patients with COVID-19 in Granada, Spain. Two visits were scheduled in the follow-up period from May to October 2020. A total of 372 patients—with 217 included and 115 excluded—were referred 2-months after diagnosis of COVID-19 for the first follow-up consultation (FFuC). The 217 patients included in the FFuC were referred to the second follow-up consultation (SFuC) at 6-months after initial diagnosis—with 207 included and 10 excluded. For COVID-19 detection, was used the RT-PCR from the upper respiratory tract (nasopharyngeal and oropharyngeal swab) or lower respiratory tract (sputum collection) and antibody serology (IgM and IgG) by ELISA. The study timeline, flowchart, and follow-up consultation procedures are shown in Fig. 1. The study was conducted following the requirements of the Declaration of Helsinki and the Spanish Data Protection Act of 15/1999. Following the Declaration of Helsinki, written informed consent was obtained from all patients, and local ethics committees approved the study.

The study population consisted of patients aged > 14 years, diagnosis of infection confirmed according to international recommendations and signing the informed consent. The exclusion criteria were: suspected cases of SARS-CoV-2, immunosuppressed patients with therapeutic limitation due to terminal pathology, and those who refused to participate (see Fig. 1). The age, sex, BMI, toxic habits, profession, family members with confirmed or suspected COVID-19, and need or not of hospital admission were considered. See Table 2, which summarizes the characteristics of the patients included in the study.

### Data collected in follow-up consultations

The clinical features studied at each follow-up visit were: dry and wet cough, dyspnea, fatigue, muscle weakness, musculoskeletal involvement, chest pain, palpitations, fever, sweating, intestinal disorders, post-COVID-19 arterial hypertension, otorhinolaryngologic symptoms, ocular symptoms, neurological manifestations, decreased sexual appetite, cutaneous manifestations and other expressed symptoms—thromboembolic events. Also, each consultation included mental abnormalities, physical examination, and laboratory indices (see Tables 1, 2).

**Table 1:** Comparative table of the laboratory reference ranges considered normal in the adult population according to the international system and the provincial area of Granada.

Test in the study population (units)	Reference ranges	
	Laboratory-UH-VN	References^29,42,43^
**Biochemical**		
Glucosa (mmol/L)	4.16–6.38	3.9–6.1
Total bilirubin (µmol/L)	5.13–20.52	0–26
Creatinine (µmol/L)	59.23–106.08	57–111
Alanine aminotransferase (ALT, SGPT) (U/L)	7–34	10–40
Aspartate aminotransaminase (AST, SGOT) U/L	10–35	10–40
Lactato deshidrogenasa (LDH), U/L	0–247	0–245
Serum ferritin (ng/mL)	10–120	21–274.66
Total protein (g/dL)	6.6–8.3	6.5–8.5
**Hematologic**		
Haemoglobin (g/dL)	12–15.6	13–17.5
Leukocytes (10^9^ L)	3.9–10.2	3.5–9.5
Neutrophils (%)	42–77	50–70
Lymphocytes (%)	20–44	30–45
Lymphocytes (× 10^9^ L)	1.1–4.5	1.1–3.2
Platelets (× 10^9^ L)	130–370	125–359
**Coagulation function**		
d-Dimer **(**μg/L)	0–0.5	0–1

Laboratory reference ranges and characteristics of the patients included in the study.

**Table 2:** Summary of the characteristics of the patients included in the study.

**Characteristics (**N = 217**)**
Age (years), median (IQR)
Gender, (N (%))
Female and Male
BMI (kg/m2)
Smoking history, (N° (%)): Cumulative tobacco burden index (ICAT), Current, Former and Nonsmoker
Healthcare professional (N° (%))
Need or not of hospital admission (N° (%))
**First follow-up consultations (**N = 217**)**
Clinical features
Asymptomatic (N° (%))
Any one of the following symptoms N° (%): Dyspnea, Fatigue, Cough (dry and wet), Muscle weakness, Arthralgia or Myalgia, Chest pain, Palpitations, Fever (temperature ≥ 37.3 °C), Sweating, N° (%)
Intestinal disorders (N° (%)): Nausea, Vomiting, Diarrhoea
Post covid arterial hypertension**,** N° (%)
Otorhinolaryngologic symptoms (N° (%)): Rhinorrhea, difficulty to swallow, tinnitus, nasal dryness, dysphonia, and hearing loss
Loss of taste or smell, dizziness, and gait instability (N° (%))
Ocular symptoms (N° (%))
Epiphora and reduced visual acuity (N° (%))
Neurological Manifestations (N° (%)): Polyneuro/myopathy, Headaches, Cognitive deficits
Erectile dysfunction and decreased sexual apetite (N° (%))
Cutaneous manifestations (N° (%)): Skin rash, Rash skin eruptions, Hair loss
Thromboembolic events (N° (%)) and types thromboembolic events
Mental health
Emotional affectation, Depression, and Sleep disturbance (N° (%))
Physical examination
Lung auscultation, crackles, Sat02, nail clubbing (N° (%))
Abnormal finding on X-ray, (N° (%))
Laboratory finding (Table 1)
**Second follow-up consultations (N = 207)**
Clinical features, mental health, physical examination, X-ray, and laboratory tests as in the FFuC
Pre-existing respiratory disease (PRD) (N° (%))
Pulmonary function
FVC < 80%, FEV1 < 80%, FEV1/FVC < 70%, TLC < 80%, VR < 65%, DLCO < 80% and KCO < 80% (% of predicted)
6MWT
Distance-meters, median (IQR) and oxygen saturation, median (Initial, Final, and Average) (IQR)
Chest CT
Density (Nº (%)): Mixed pattern, Consolidation, and Ground-glass
Location (Nº (%)): Peripheral Central and Mixed
Subpleural reticular pattern (Nº (%)): Interlocular septal thickening and thickening of the adjacent pleura
Lung involvement (Nº (%)): Unilateral and Bilateral
Bronchiectasis (Nº (%))
Others findings of CT (Nº (%))

In all patients, a chest radiograph was performed in posterior-anterior and lateral projection at the FFuC. Then, a complementary study with HRCT was requested if there were any abnormal findings on the X-ray. HRCTs were evaluated by radiologist specialists and one pulmonologist and reported according to the Spanish Society of Medical Radiology (SERAM) recommendations, the international standard nomenclature defined by the Fleischner Society glossary and existing publications until now. Each imaging test was analyzed considering: density (ground glass, consolidation or mixed), lung involvement (unilateral or bilateral), location (central, peripheral or mixed), presence of reticular pattern or interstitial lesions of pulmonary parenchymal (subpleural or interlobular), and the percentage of lung extension involved < 20%, 20–50% and > 50%—according to the lung fields involved (see Table 2).

At the SFuC, each patient underwent forced spirometry, lung volume, diffuse capacity, and the 6MWT. The functional exploration was carried out with experienced personnel with the equipment of MasterScreen Body, brand Jaeger, Germany. According to American Thoracic Society and Spanish regulations, the reference values for the Mediterranean population and acceptability criteria. Pulmonary parameters included FEV1, FVC, the FEV1/FVC ratio, RV, TLC, DLCO, and KCO.

### Statistical analysis

Descriptive analysis was carried out using number (%), median, and its interquartile range (IQR)—combining box plots and density plots, i.e., violin plots—for categorical and continuous variables, respectively^39,40^. Discrepancies in the patient characteristic distributions by sub-groups of outcomes are presented as differences with 95% confidence intervals (CIs). The Mann–Whitney U test—for nonnormal distributed continuous data, χ^2^ test, or Fisher's exact was used to compare clinical features, physical examination, mental health, and laboratory indices between males and females at the first and second follow-up consultations.

Bivariate and multivariate analysis—using the maximum likelihood estimation to obtain the coefficients and the of Hosmer–Lemeshow goodness-of-fit test for the mode—was carried out to compute the odds ratios (ORs) and 95% CIs to explore the association with the following features: at least one pre-existing respiratory disease, hospitalized patients, and an abnormal chest CT finding^41^. The degree of association of the PCS is defined according to the OR value (robust or not). Data cleaning and analysis using logistic regression models were implemented in Python 3.7. The tests were two-sided, and a p value less than α = 0.05 was considered statistically significant.

## Supplementary Information

Supplementary Information.

## Data Availability

Currently, the third follow-up consultation (one year after the disease) is being collected, which means that data cannot be shared. Once the data analysis process for each follow-up process is completed, the data and the implemented code could be shared on an internet hosting, such as GitHub repository.

## Abbreviations

SARS-CoV-2: Severe acute respiratory syndrome coronavirus 2
SARS-1: Severe acute respiratory syndrome
MERS: Middle East Respiratory Syndrome
PCS: Post-COVID-19 syndrome
ICAT: Coalition Against Tobacco
BMI: Body mass index
FFuC: First follow-up consultation
SFuC: Second follow-up consultation
X-Ray: Chest radiograph
CT: Chest computed tomography
HRCT: High-resolution computed tomography
GGO: Ground glass opacity
DILD: Diffuse interstitial lung disease
PFT: Pulmonary function test
FEV1: Forced expiratory volume in the first second
FVC: Forced vital capacity
FEV1/FVC: The FEV1/FVC ratio
RV: Residual volume
FRC: Residual functional capacity
TLC: Total lung capacity
DLCO: Carbon monoxide transfer by a single breath
KCO: Diffusion constant for carbon monoxide
6MWT: 6-Minute walking test
SERAM: Spanish Society of Medical Radiology
IQR: Interquartile range
CIs: Confidence intervals
ORs: Odds ratios
BVA: Bivariate analysis
MVA: Multivariate analysis
PRD: Pre-existing respiratory disease
RT-PCR: Reverse transcription-polymerase chain reaction
PE: Pulmonary embolism
