# Supplementary material for: On the single and multiple associations of COVID-19 post-acute sequelae: 6-month prospective cohort study
Source: Sci Rep. 2022 Mar 1;12:3402. doi: 10.1038/s41598-022-07433-8 (PMC8888754; doi:10.1038/s41598-022-07433-8)
Supplement: Supplementary file 1 — Supplementary Information. [file 41598_2022_7433_MOESM1_ESM.docx]

# Supplementary Materials for

# On the single and multiple associations of COVID-19 post-acute sequelae: 6-month prospective cohort study.

## Beatriz María Jiménez-Rodríguez*,†,1,2, José Gutiérrez-Fernández2,3,4, Eldis Maria Ramos-Urbina1, Ana Dolores Romero-Ortiz1,4, Paula Isabel García-Flores1, Maria Inmaculada Santiago-Puertas1, Maria José Martín-López1 , Genaro López-Milena5, Rene Fabregas*,†,6,7 and Concepción Morales-García*,†,1,4

*1Department of Pneumology, University Hospital Virgen de las Nieves, Granada, Spain.*

*2Department of Microbiology, School of Medicine and PhD Program in Clinical Medicine and Public Health, University of Granada-IBS, 18010, Granada, Spain.*

*3Department of Microbiology, University Hospital Virgen de las Nieves, Granada, Spain.*

*4Biosanitary Research Institute of Granada-ibs, Granada, Spain.*

*5Department of Radiodiagnosis, University Hospital Virgen de las Nieves, Granada, Spain.*

*6Department of Applied Mathematics and Research Unit ``Modeling Nature'' (MNat), Faculty of Sciences, University of Granada, 18071 Granada, Spain.*

*7Department of Physics and Astronomy, University of Manchester, Manchester M13 9PL, UK*

†Equally Contributing Authors. *Corresponding Authors: [beajirod@gmail.com,](mailto:beajirod@gmail.com) [rfabregas@ugr.es](mailto:rfabregas@ugr.es) and [concepcion.morales.sspa@juntadeandalucia.es](mailto:concepcion.morales.sspa@juntadeandalucia.es)

**S1. Clinical features, physical examination, psychology affectations, and laboratory indices.**

**Figure S1** shows a descriptive analysis of gender, physical examination, mental health, and laboratory indices for the first and second follow-up consultations. Note here that the violin plots clearly show the degree of severity in the laboratory results.

| 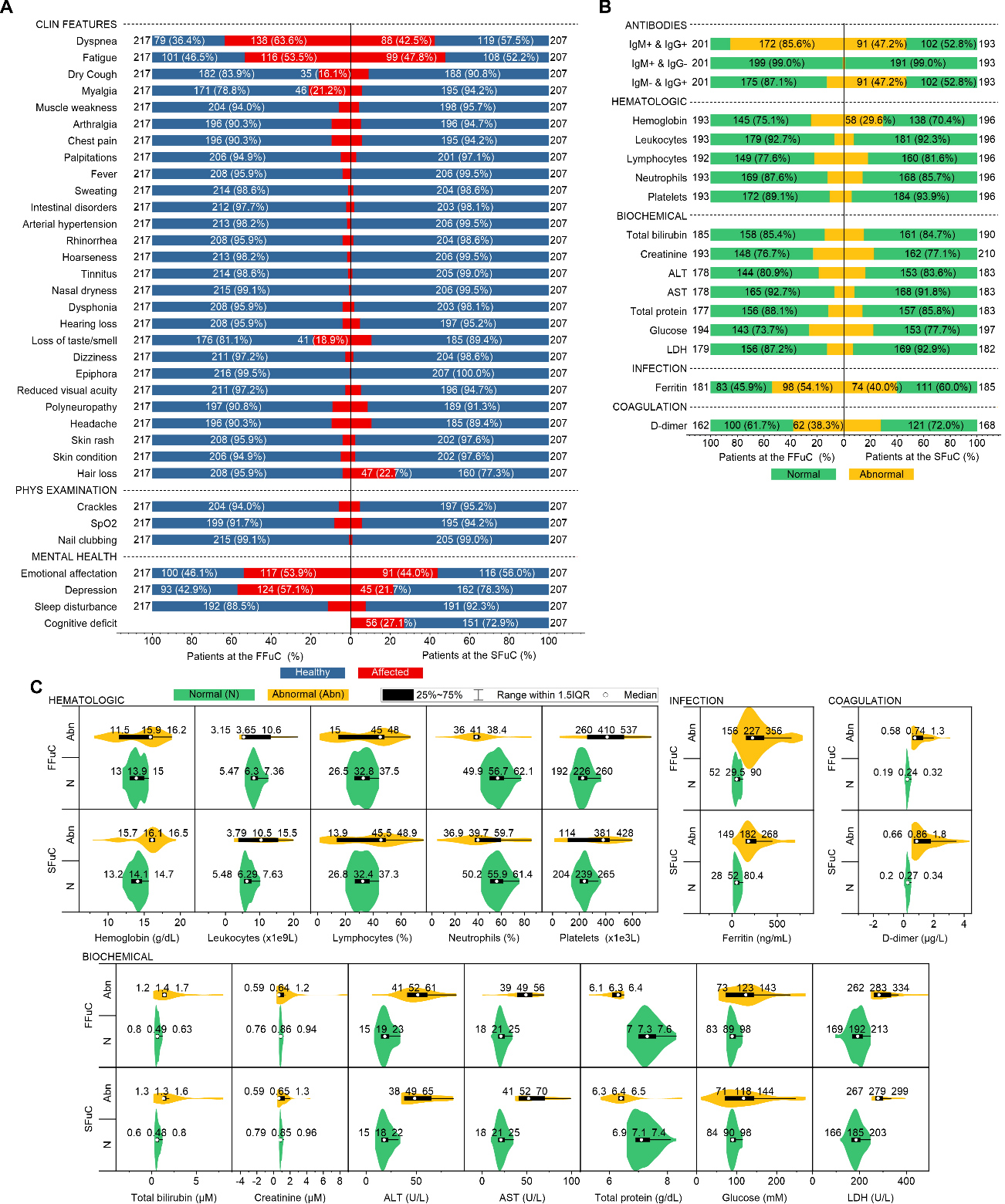 |
| --- |
| **Figure S1.** **(A)** Clinical features, physical examination, and mental health of patients with PCS for first/second follow-up consultations. **(B)** Frequency presented in Nº (%) for the laboratory indices of patients with PCS for first/second follow-up consultations. **(C)** Violin plots show the distribution, median, and quartiles of the laboratory indices for first/second follow-up consultations. |

**S2-S3. Pre-existing respiratory disease and hospitalization.**

**Figure S2** and **S3** show a descriptive analysis for gender, demographic characteristics, clinical outcomes, respiratory function tests, and mental health for patients with and without a PRD, as well as hospitalized and non-hospitalized.

| 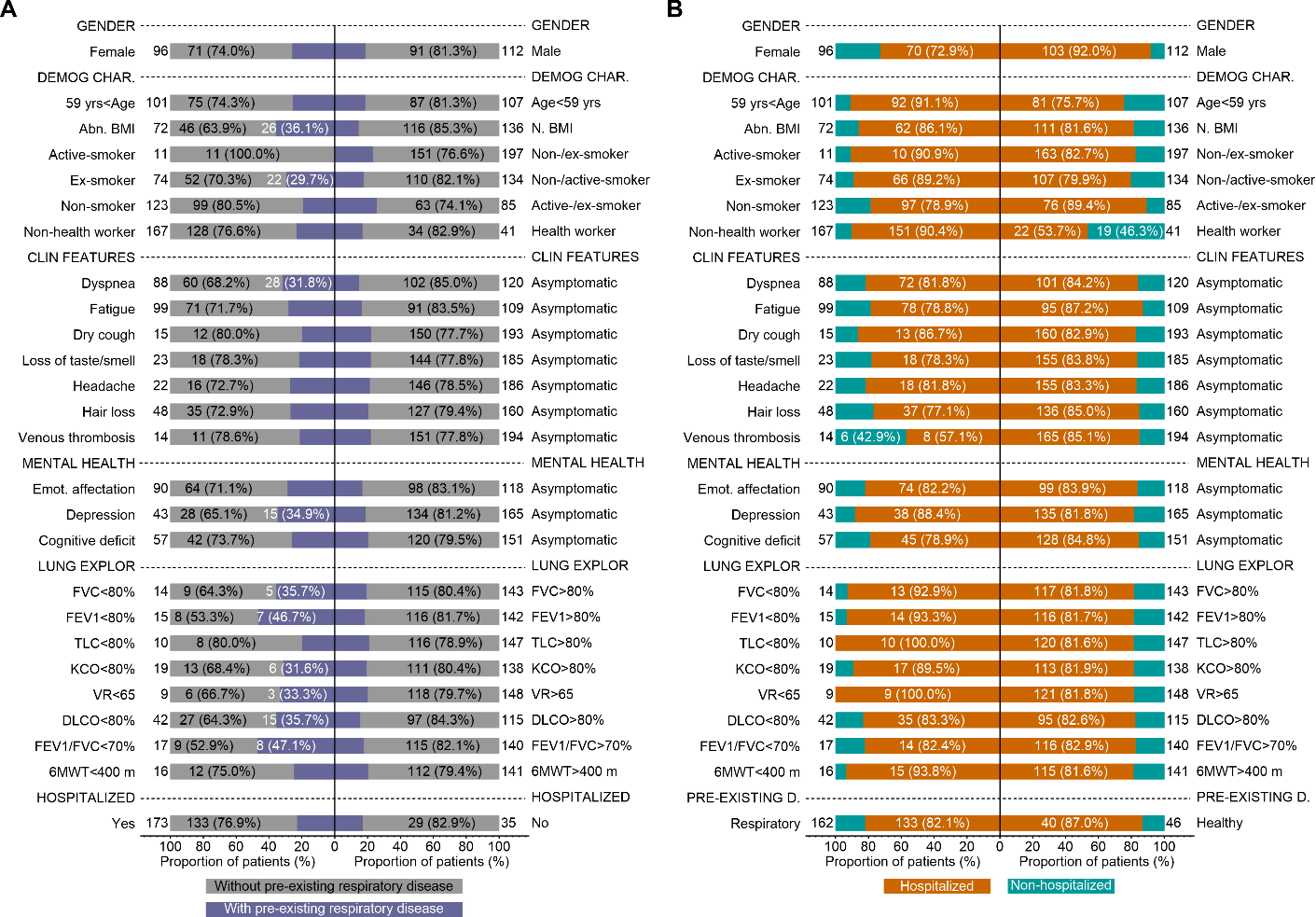 |
| --- |
| **Figure S2.** Frequency presented in Nº (%) of patients by gender, clinical and demographic characteristics, mental health impairment, and pulmonary function tests with/without a pre-existing respiratory disease **(A)** and hospitalized/non-hospitalized **(B)**. |

| 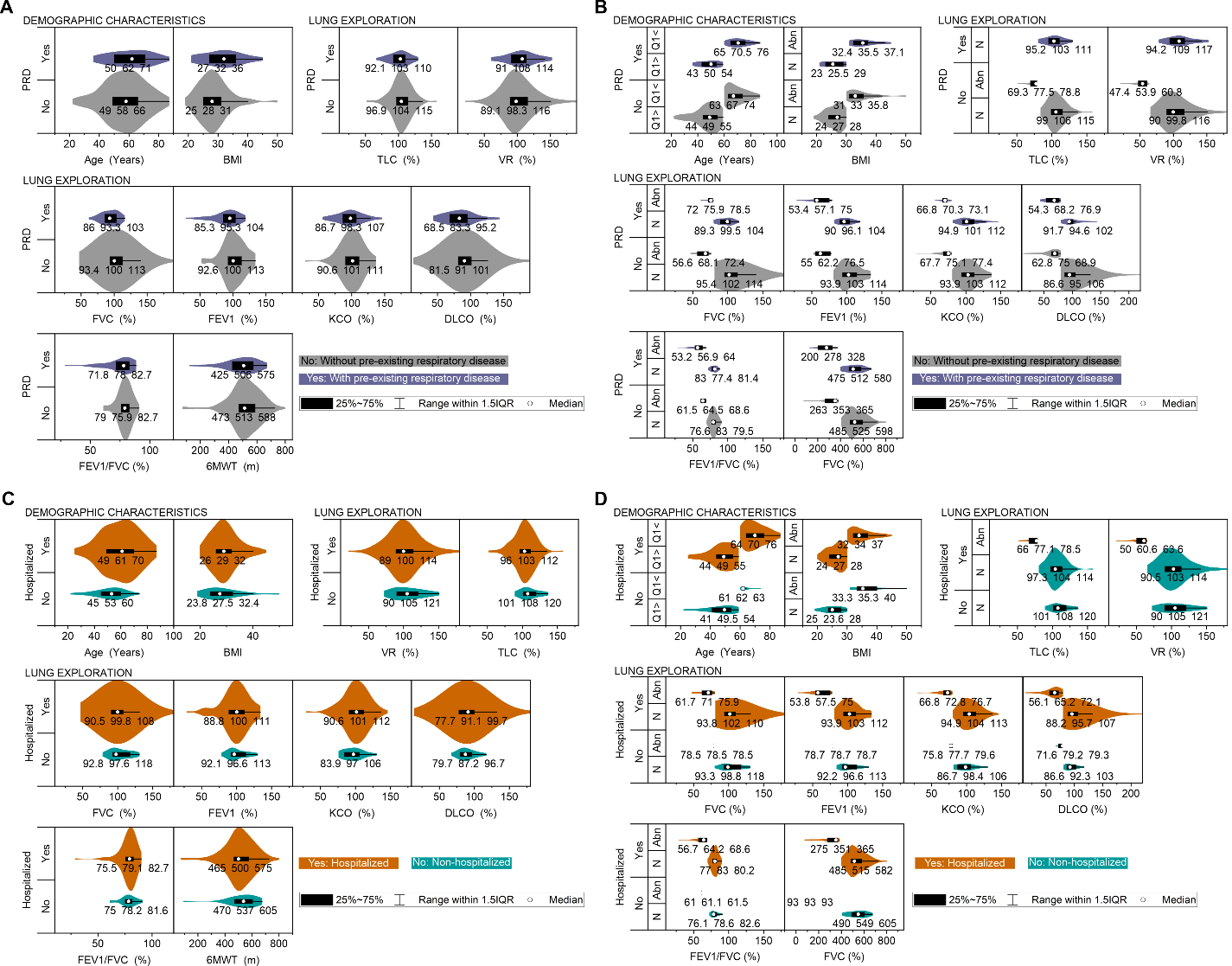 |
| --- |
| **Figure S3.** Violin plots show the distribution, median, and quartiles of the laboratory indices for first and second follow-up consultations of patients with/without a pre-existing respiratory disease **(A)**-**(B)**, and hospitalized/non-hospitalized **(C)**-**(D)**. |

**S4. Chest high-resolution CT scan findings and laboratory reference ranges.**

| 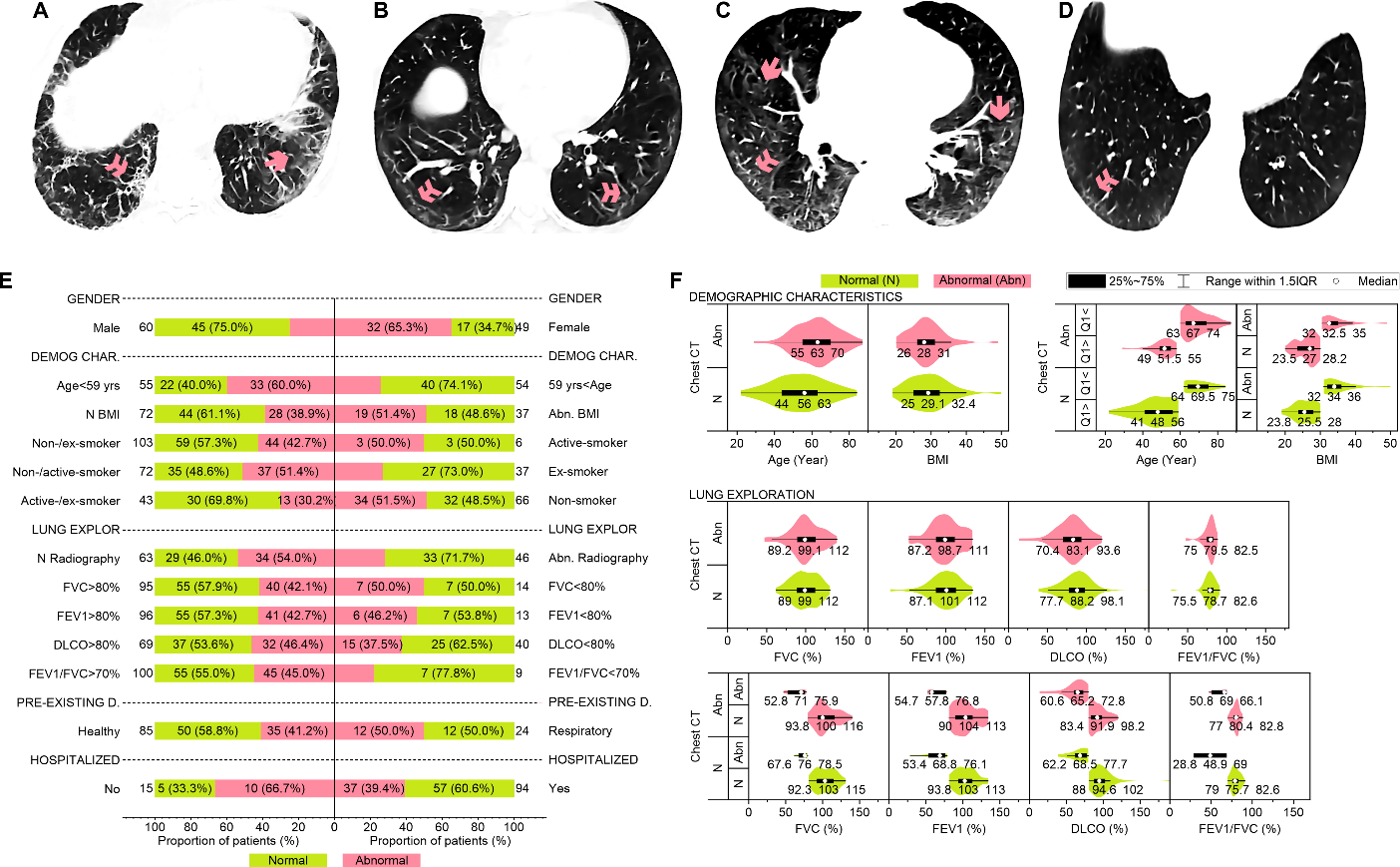 |
| --- |
| **Figure S4.** CT scan images of 4 patients **(A)**-**(C)**, and findings in all patients at the 6-month follow-up. **(E)** Stacked bars showed the frequency in Nº (%) of patients for each feature with normal (green color) and abnormal (pink color) CT. **(F)** Violin plots show each demographic characteristic's distribution, medians, and IQR and lung exploration test. |

**Figures S4A-B** show the CT scans images of 4 patients after 6-months of follow-up. A 66-year-old man — BMI=32.8 and with PRD — with peripherally distributed GGOs (single arrow) and reticular pattern with architectural distortion of the parenchyma and focal traction bronchiectasis (double arrow) (**Fig. S4A**). A 58-year-old woman — BMI=28 and without PRD — with subpleural bands in both lungs, predominantly bibasal and primarily right (double arrow) (**Fig. S4B**). A 74-year-old man — BMI=30.1 and without PRD — with diffuse GGOs (single arrow) and reticular pattern with thickening of peripherally distributed interlobular septal (double arrow) (**Fig. S4C**). A 70-year-old man — BMI=30.4 and without PRD — with slight peripheral subpleural reticulation with small bronchiectasis (double arrow) (**Fig. S4D**). **Figures S4E-F** display a descriptive analysis of gender, demographic characteristics, lung function tests, PRD conditions, and hospitalized status.
